# Supplementary material for: Enhancing generalizability of model discovery across parameter space with multi-experiment equation learning for biological systems
Source: PLoS Comput Biol. 2026 Apr 22;22(4):e1014161. doi: 10.1371/journal.pcbi.1014161 (PMC13132452; doi:10.1371/journal.pcbi.1014161)
Supplement: S1 Text — Details on methods to calculate the optimal regularization hyperparameter λ and the procedure used to select the final model. (PDF) [file pcbi.1014161.s001.pdf]

S1 Text: Calculating the optimal regularization hyperparameter λ

When optimizing $\hat{\xi}$ in Eq. 7, the hyperparameter λ governs the tradeoff between model complexity and fit. Since we do not know *a priori* how complex the model should be, we perform cross-validation to determine the optimal regularization parameter, λ, for the LASSO algorithm, using the pySINDy package for SINDy implementation [1, 2].

We perform a grid search over $\lambda_j = 10^{-1}, ..., 10^{-9}$ with 100 equi-log-spaced values. For each value λ_j, we randomly select 10 train-test splits (each with the standard 80% training and 20% testing data) of $\frac{dC_d(t)}{dt}$. For each split, we solve Eq. 7 using LASSO with the specified library to obtain the optimal coefficients $\hat{\xi_k}, k = 1, ..., 10$. For each optimal coefficient $\hat{\xi_k}$, we then forward solve Eq. 6 and calculate the sum of squared errors (SSE) between the forward solution of the learned differential equation model and the test data. Using this information, we calculate the Akaike Information Criteria (AIC) score for each train-test split. We note that using AIC or variants such as the corrected AICc for model selection is an established practice in the literature [3]. Since our dataset is not sparse in time, the correction is not necessary and we use the standard AIC score.

Each value λ_j is quantified with a final AIC score which is the average AIC over all 10 train-test splits. Once all AIC values are calculated for all λ, we select the λ_j for which the mean AIC is minimal. However, we found that when λ was too small, selected models had many nonzero terms with large non-interpretable coefficients. The smaller the value of λ, the larger the oscillating coefficients. Therefore, we defined the *lower bound* for the optimal λ as the λ with the minimum AIC score.

Because we do not expect large coefficients due to our domain knowledge, we require that the optimal λ selected must have every recovered model in the train-test split have all model coefficients below a threshold value, which we set to 100 for OAT ME-EQL and 20 for ES ME-EQL (these two values correspond to each other because in the ES ME-EQL approach, each coefficient is multiplied by R_p, which varies between 0 and 5). If we find that the value of λ with the minimum AIC score contained at least one model for which any coefficient is above the specified threshold, we increased the value of λ until we find one that met this criterion.

We display an example plot of $\bar{\lambda}$ and AIC values in S1 Fig. for $R_p = 1$ (left) and $R_p = 5$ (right). For each case, the plots appear step-like, indicating different model structures are being selected at each jump in the plot (labeled with which terms are in the final selected model structure). Note that the final selected λ is not always the one that generates the lowest mean AIC score, but instead must also satisfy the second criteria in which none of the learned coefficients can be greater in magnitude than 100 for any of the 10 test-train splits.

Selecting the final model

Once the optimal λ is selected, we examine the 10 test-train splits to determine the most popular learned model structure. Of the 10 models that contain the most popular learned model structure, we average the coefficient values to find $\bar{\xi}$. To find the final model coefficients ξ_f, we re-optimize the parameters to the most popular learned model structure using all of the data. We use a Nelder-Mead algorithm and $\bar{\xi}$ is used as the initial parameter guess.

References

[1] Brian M. de Silva, Kathleen Champion, Markus Quade, Jean-Christophe Loiseau, J. Nathan Kutz, and Steven L. Brunton. PySINDy: A Python package for the sparse identification of nonlinear dynamical systems from data. Journal of Open Source Software, 5(49):2104, 2020.

[2] Alan A. Kaptanoglu, Brian M. de Silva, Urban Fasel, Kadierdan Kaheman, Andy J. Goldschmidt, Jared Callaham, Charles B. Delahunt, Zachary G. Nicolaou, Kathleen Champion, Jean-Christophe Loiseau, J. Nathan Kutz, and Steven L. Brunton. PySINDy: A comprehensive Python package for robust sparse system identification. Journal of Open Source Software, 7(69):3994, 2022.

[3] Niall M. Mangan, J. Nathan Kutz, Steven L. Brunton, and Joshua L. Proctor. Model selection for dynamical systems via sparse regression and information criteria. Proceedings of the Royal Society A, 473(2204):20170009, 2017.
