# Supplementary material for: Enhancing generalizability of model discovery across parameter space with multi-experiment equation learning for biological systems
Source: PLoS Comput Biol. 2026 Apr 22;22(4):e1014161. doi: 10.1371/journal.pcbi.1014161 (PMC13132452; doi:10.1371/journal.pcbi.1014161)
Supplement: S2 Text — Algorithms for the OAT ME-EQL and ES ME-EQL approaches. (PDF) [file pcbi.1014161.s002.pdf]

S2 Text: Algorithmic differences between OAT ME-EQL and ES ME-EQL

The one-at-a-time (OAT) and embedded structure (ES) variants of ME-EQL differ in how they handle data across parameter values during both hyperparameter tuning and model selection. A detailed description of the methodology for hyperparameter tuning and model selection can be found in the Supplementary Material, and the corresponding algorithms for each of the two equation learning approaches are summarized in Algorithms 1 and 2 below. In the OAT approach, each dataset corresponding to a specific parameter value is treated independently: for each R_p, a separate grid search over λ is performed, leading to potentially different optimal regularization parameters and selected models across parameter settings. After model selection via AIC-based majority vote, each model is re-optimized individually and the final OAT ME-EQL model is constructed by interpolating coefficients across the most commonly-retained models. In contrast, the ES ME-EQL approach treats the entire set of datasets jointly by embedding parameter values directly into the function library. As a result, a single global λ is selected through cross-validation over all parameterized datasets, and the model structure is determined via majority vote across the aggregate train-test splits. This unified treatment enforces structural consistency across the parameter space and produces a single, global model fit to all the data. Thus, OAT ME-EQL is better suited for uncovering local structures and heterogeneity, while ES ME-EQL emphasizes global coherence and structural generalizability.

Algorithm 1 One-at-a-Time (OAT) ME-EQL Model

Input:
    Set of parameters $P = \{R_p\}$
    Data for each parameter: $D = \left\{C_{d,R_p}\right\}$
    Regularization set: $\Lambda = \{\lambda_j\}$
Final Output: OAT Model
for all $R_p \in P$ **do**
    for all $\lambda_j \in \Lambda$ **do**
        Determine $\hat{\xi}_{R_p,\lambda_j}$ by minimizing Equation (7) in the main text
        Record AIC score
    end for
    Select λ^* with minimum AIC score and no learned coefficient magnitudes exceeding 100.
    Select model structure by majority vote across test-train splits.
    Re-optimize model parameters using data for R_p.
end for
Retain R_p values and optimized models with the majority learned structure.
Determine final OAT-model by interpolating coefficients for each term in the majority model over R_p values.

Algorithm 2 Embedded Structure (ES) ME-EQL Model

Input:
  Set of parameters $P = \{R_p\}$
  Data for all parameters: $D = \left\{[C_{d,R_{p_1}}, C_{d,R_{p_2}}, \ldots, C_{d,R_{p_N}}]\right\}$
  Regularization set: $\Lambda = \{\lambda_j\}$
Final Output: ES Model
for all $\lambda_j \in \Lambda$ **do**
  Determine $\hat{\xi}_{\lambda_j}$ by minimizing minimizing Equation (7) in the main text
  Record AIC score
end for
Select λ^* with minimum AIC score and no learned coefficient magnitudes exceeding 20.
Select model by majority vote across test-train splits.
Determine final ES-model: Re-optimize using D.
