# Supplementary material for: Enhancing generalizability of model discovery across parameter space with multi-experiment equation learning for biological systems
Source: PLoS Comput Biol. 2026 Apr 22;22(4):e1014161. doi: 10.1371/journal.pcbi.1014161 (PMC13132452; doi:10.1371/journal.pcbi.1014161)
Supplement: S1 Fig — Optimal λ selected in red square. Text indicates the learned model structure at each jump in the plot. (PDF) [file pcbi.1014161.s003.pdf]

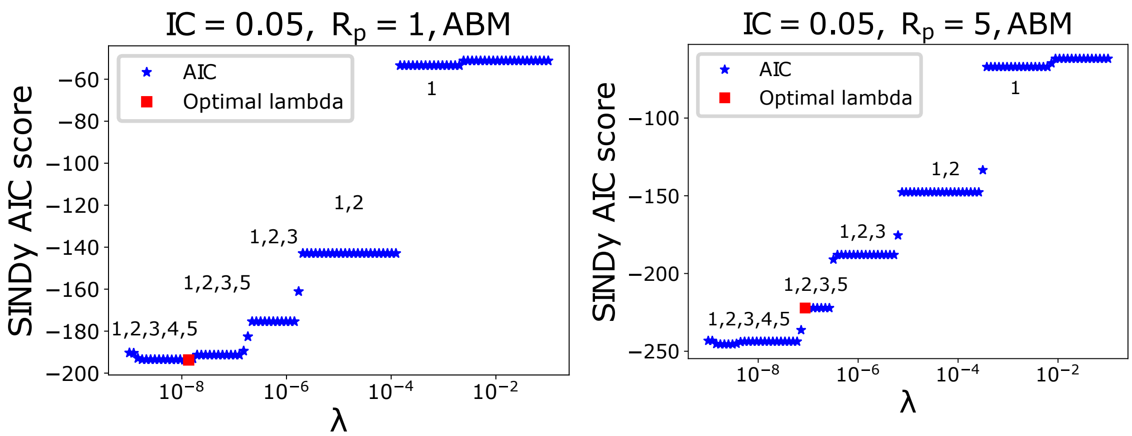

S1 Fig.: Example plots of the hyperparameter $\bar{\lambda}$ plotted against the AIC scores of $R_p = 1$ (left) and $R_p = 5$ (right). Optimal λ selected in red square. Text indicates the learned model structure at each jump in the plot.
