# Supplementary material for: Enhancing generalizability of model discovery across parameter space with multi-experiment equation learning for biological systems
Source: PLoS Comput Biol. 2026 Apr 22;22(4):e1014161. doi: 10.1371/journal.pcbi.1014161 (PMC13132452; doi:10.1371/journal.pcbi.1014161)
Supplement: S2 Fig — Snapshots of ABM simulations of birth, death, and migration dynamics at different timepoints for initial conditions with 5% and 25% of sites occupied, respectively, and corresponding mean population sizes in time across 25 ABM simulations. (PDF) [file pcbi.1014161.s004.pdf]

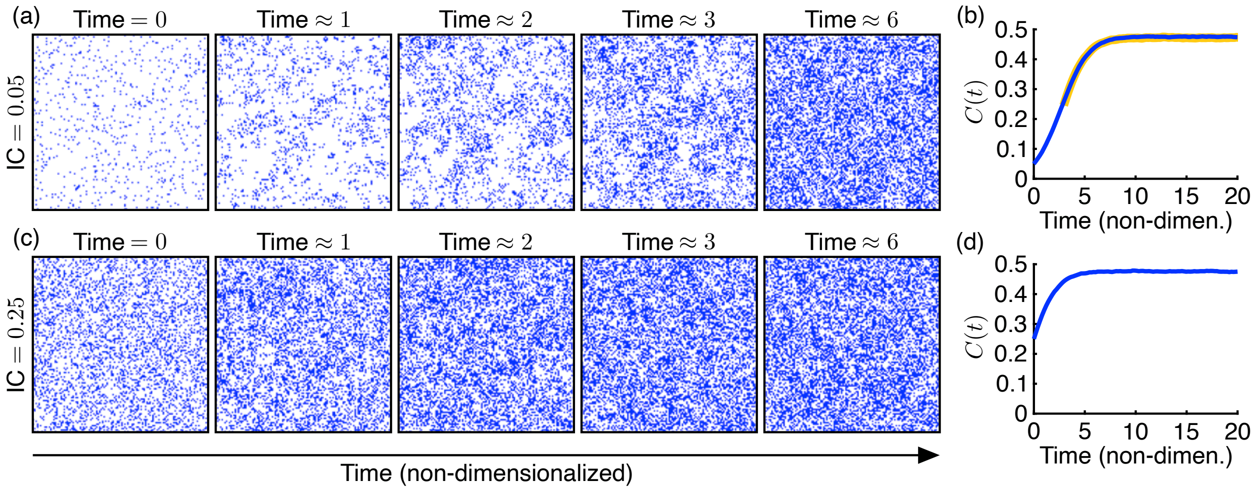

S2 Fig.: Example model simulations highlighting how initial conditions affect information content. Panels (a) and (c) each present snapshots of an ABM simulation of birth, death, and migration dynamics at different timepoints for initial conditions with 5% and 25% of sites occupied, respectively. (Blue denotes occupied sites, while white denotes unoccupied sites.) Similarly, panels (b) and (d) show the corresponding mean population sizes in non-dimensionalized time for IC $= 0.05$ and IC $= 0.25$, respectively, averaged across 25 ABM simulations each. Here we set $R_p = 0.1$, $R_d = 0.05$, and $R_m = 1$, and consider a 120×120 square lattice. The yellow curve in panel (b) is a copy of the mean population-size curve from panel (d), shifted in time and shortened to roughly align with the latter portion of the $C(t)$ curve in panel (b). This yellow curve serves to highlight that changing the ABM initial conditions allows us to control the amount of information content in our data. In particular, using IC $= 0.25$ effectively means that we observe less of the ABM time dynamics than we do when IC $= 0.05$.
