# Supplementary material for: Enhancing generalizability of model discovery across parameter space with multi-experiment equation learning for biological systems
Source: PLoS Comput Biol. 2026 Apr 22;22(4):e1014161. doi: 10.1371/journal.pcbi.1014161 (PMC13132452; doi:10.1371/journal.pcbi.1014161)
Supplement: S1 Table — Models learned using ME-EQL methods for the mean-field model data with initial conditions 0.05 and 0.25, and for noise levels σ=0% and σ=0.25%. (PDF) [file pcbi.1014161.s005.pdf]

S1 Table.: Models learned using ME-EQL methods for the mean-field model data with initial conditions 0.05 and 0.25, and for noise levels $\sigma = 0\%$ and $\sigma = 0.25\%$. Coefficients are displayed and rounded to at most 3 decimal places. The bolded equation is the ground-truth, correct mean-field model.

**Setting**	**Experiments used**	**OAT ME-EQL**	**ES ME-EQL**
IC = 0.05, $\sigma = 0\%$	500	$\boldsymbol{dC/dt = 0.50 R_p C - 1.00 R_p C^2}$	$dC/dt = 0.50R_pC - 1.00R_pC^2$
	10	$dC/dt = 0.50R_pC - 1.00R_pC^2$	$dC/dt = 0.50R_pC - 1.00R_pC^2$
	5	$dC/dt = 0.50R_pC - 1.00R_pC^2$	$dC/dt = 0.50R_pC - 1.00R_pC^2$
IC = 0.05, $\sigma = 0.25\%$	500	$dC/dt = 0.50R_pC - (1.00R_p + 0.001)C^2$	$dC/dt = 0.50R_pC - 1.02R_pC^2 + 0.02R_pC^3$
	10	$dC/dt = 0.499R_pC - (0.998R_p - 0.001)C^2$	$dC/dt = 0.50R_pC - 1.00R_pC^2$
	5	$dC/dt = (0.5R_p - 0.002)C - (1.001R_p - 0.005)C^2$	$dC/dt = 0.50R_pC - 1.00R_pC^2$
IC = 0.25, $\sigma = 0\%$	500	$dC/dt = 0.50R_pC - 1.00R_pC^2$	$dC/dt = 0.49R_pC - 0.97R_pC^2 - 0.03R_pC^3$
	10	$dC/dt = 0.50R_pC - 1.00R_pC^2$	$dC/dt = 0.5R_pC - 0.98R_pC^2 - 0.02R_pC^3$
	5	$dC/dt = 0.50R_pC - 1.00R_pC^2$	$dC/dt = 0.5R_pC - 1.02R_pC^2 + 0.03R_pC^3$
IC = 0.25, $\sigma = 0.25\%$	500	$dC/dt = 0.501R_pC - (1.001R_p + 0.001)C^2$	$dC/dt = 0.50R_pC - 1.01R_pC^2 + 0.01R_pC^3$
	10	$dC/dt = (0.50R_p + 0.003)C - (1.001R_p + 0.006)C^2$	$dC/dt = 0.51R_pC - 1.04R_pC^2 + 0.05R_pC^3$
	5	$dC/dt = (0.508R_p - 0.002)C - (1.016R_p - 0.004)C^2$	$dC/dt = 0.51R_pC - 1.05R_pC^2 + 0.06R_pC^3$
