# Supplementary material for: Enhancing generalizability of model discovery across parameter space with multi-experiment equation learning for biological systems
Source: PLoS Comput Biol. 2026 Apr 22;22(4):e1014161. doi: 10.1371/journal.pcbi.1014161 (PMC13132452; doi:10.1371/journal.pcbi.1014161)
Supplement: S2 Table — Models learned using ME-EQL methods for the ABM data with initial conditions 0.05 and 0.25. (PDF) [file pcbi.1014161.s006.pdf]

S2 Table.: Models learned using ME-EQL methods for the ABM data with initial conditions 0.05 and 0.25. Coefficients are displayed and rounded to at most 3 decimal places.

**Setting**	**Experiments used**	**OAT ME-EQL**	**ES ME-EQL**
IC = 0.05	500	$dC/dt = (-0.003R_p^3 + 0.023R_p^2 + 0.29R_p + 0.067)C^1$<br>$+(0.06R_p^3 - 0.603R_p^2 - 1.286R_p - 0.409)C^2$<br>$+(-0.215R_p^3 + 2.263R_p^2 + 1.182R_p + 1.116)C^3$<br>$+(0.538R_p^3 - 6.3R_p^2 + 2.262R_p - 3.016)C^5$	$dC/dt = 0.36 * R_pC - 2.34R_pC^2$<br>$+5.6R_pC^3 - 5.0R_pC^4$
	10	$dC/dt = (0.006R_p^3 - 0.038R_p^2 + 0.415R_p - 0.0005)C^1$<br>$+(-0.075R_p^3 + 0.372R_p^2 - 3.286R_p + 0.665)C^2$<br>$+(0.261R_p^3 - 1.142R_p^2 + 8.09R_p - 2.525)C^3$<br>$+(-0.739R_p^3 + 2.777R_p^2 - 15.855R_p + 6.165)C^5$	$dC/dt = 0.4R_pC - 2.53R_pC^2$<br>$+5.81R_pC^3 - 4.92R_pC^4$
	5	$dC/dt = (-0.025R_p^3 + 0.186R_p^2 - 0.065R_p + 0.29)C^1$<br>$+(0.22R_p^3 - 1.769R_p^2 + 1.202R_p - 1.896)C^2$<br>$+(-0.516R_p^3 + 4.451R_p^2 - 3.397R_p + 3.707)C^3$<br>$+(0.716R_p^3 - 7.55R_p^2 + 4.693R_p - 4.042)C^5$	$dC/dt = 0.42R_pC - 2.37R_pC^2$<br>$+3.92R_pC^3 - 3.84R_pC^5$
IC = 0.25	500	$dC/dt = (0.004R_p^3 + 0.074R_p^2 + 0.79R_p - 0.197)C^1$<br>$+(-0.008R_p^3 - 0.423R_p^2 - 2.652R_p + 0.886)C^2$<br>$+(0.004R_p^3 + 1.486R_p^2 + 4.028R_p - 2.12)C^4$	$dC/dt = 0.34R_pC - 0.85R_pC^2$
	10	$dC/dt = (-0.107R_p^3 + 0.93R_p^2 - 1.02R_p + 0.568)C^1$<br>$+(0.463R_p^3 - 4.092R_p^2 + 5.163R_p - 2.421)C^2$<br>$+(-1.235R_p^3 + 11.218R_p^2 - 16.917R_p + 6.732)C^4$	$dC/dt = 0.85R_pC - 2.95R_pC^2$<br>$+5.24R_pC^4$
	5	$dC/dt = (1.453R_p - 1.48)C^1$<br>$+(-6.041R_p + 7.39)C^2$<br>$+(15.247R_p - 23.776)C^4$	$dC/dt = 0.86R_pC - 2.92R_pC^2$<br>$+4.94R_pC^4$
